# Simulation-Based Training Improves Ophthalmology Resident Confidence in Performing YAG Capsulotomy, Peripheral Iridotomy, and Selective Laser Trabeculoplasty

**DOI:** 10.7759/cureus.107563

**Published:** 2026-04-23

**Authors:** Zaid Alsafi, Reshma Sonsale, Mostafa Dowidar, Surinder S Dosanjh, Ahmed Al-Nahrawy, Cordelia McKechnie, Ourania Frangouli, Rahila Zakir

**Affiliations:** 1 Ophthalmology, Western Eye Hospital, Imperial College Healthcare NHS Trust, London, GBR; 2 Ophthalmology, Whipps Cross University Hospital, London, GBR

**Keywords:** acute angle closure, medical education, phacoemulsification cataract surgery, primary open-angle glaucoma, simulation in medical education

## Abstract

Background and objective

Laser-based procedures such as yttrium aluminum garnet (YAG) capsulotomy, peripheral iridotomy (PI), and selective laser trabeculoplasty (SLT) are core competencies in ophthalmology, often involving a significant learning curve. This study assessed the impact of a structured simulation training program in improving ophthalmology residents’ confidence in performing YAG capsulotomy, PI, and SLT in the United Kingdom (UK).

Methods

A prospective study was conducted involving 40 UK ophthalmology residents in their first two years of training, none of whom had prior laser experience. Residents undertook a blended learning program, composed of e-learning modules, instructional videos, and a hands-on workshop using SimulEYE^®^ model eyes (insEYEt, LLC, Westlake Village, CA, USA) under the supervision of a consultant. Confidence was assessed before and after training using a 10-point Likert scale. Descriptive statistics and paired Student’s t-tests were used to compare pre- and post-training scores.

Results

Thirty-nine residents completed both pre- and post-training surveys. Following the intervention, resident confidence significantly increased across all three procedures (p < 0.0001). For YAG capsulotomy, the mean confidence score increased from 4.41 to 6.74. Confidence in performing PI rose from 2.82 to 8.21, while confidence in SLT improved from 2.41 to 7.28.

Conclusions

A structured simulation-based training program was associated with marked improvements in resident confidence across all three laser procedures, particularly for PI and SLT. The blended approach, integrating pre-course theoretical preparation with supervised hands-on simulation, offers an effective and reproducible model for early procedural training. Future studies should evaluate whether these gains are sustained over time and translate into improved clinical performance and patient outcomes.

## Introduction

Modern ophthalmology encompasses a wide range of minimally invasive and laser-based interventions. These include yttrium aluminum garnet (YAG) capsulotomy for posterior capsule opacification, peripheral iridotomy (PI) for angle-closure and narrow-angle glaucoma, and selective laser trabeculoplasty (SLT) for open-angle glaucoma. These procedures are commonly performed in outpatient settings with excellent outcomes. They are essential competencies for ophthalmologists but, like any technical intervention, involve a significant learning curve [1]. Traditionally, residents are introduced to these procedures on patients, often without prior simulation-based training. This approach may reduce confidence and increase the risk of complications, including lens pitting, uveitis, elevated intraocular pressure, hyphema, glare, and retinal detachment [1].

Simulation has emerged as a valuable adjunct to traditional patient-based teaching. It enables repetitive practice in a safe, controlled environment, offering feedback and standardization that can be difficult to achieve in clinical settings alone [2,3]. In cataract surgery, simulation has been associated with improved skill acquisition, reduced complications, and enhanced confidence [2,3]. Similar trends have also been demonstrated in other surgical specialties such as general surgery, where simulation has consistently been shown to improve confidence, reduce errors, and accelerate skill development [4].

In recent years, there has been an increased drive to expand the use of simulation within ophthalmology to support the acquisition and retention of key skills. This initiative has been strongly endorsed by the Royal College of Ophthalmologists in the United Kingdom (UK) [5]. The ideology of “see one, do one” has shifted toward structured skill acquisition in simulated environments before clinical application [6]. Notably, the EYESI surgical simulator is a prime example in ophthalmology where simulation-based training has been associated with improved real-world patient outcomes [2]. Together with the Introduction to Phacoemulsification course, these simulated programs form an essential component of the ophthalmic specialty trainee curriculum, providing residents with a foundation of skills before they progress to operating on patients [5].

Ophthalmic laser procedures, including YAG capsulotomy, PI, and SLT, are essential components of the UK ophthalmology training curriculum, with proficiency required at both Level 2 and Level 3 [7]. However, few studies have specifically evaluated the role of simulation in improving trainee confidence with these laser procedures. This study assessed the impact of a blended simulation-based training program on the confidence of UK ophthalmology residents in performing YAG capsulotomy, PI, and SLT.

## Materials and methods

This prospective study included UK ophthalmology residents in their first two years of training who had no prior experience performing ophthalmic laser procedures. The training intervention consisted of a structured blended learning program. Prior to the simulation session, participants completed mandatory e-learning modules and instructional videos covering the indications, procedural steps, laser settings, risks, and safety considerations for YAG capsulotomy, PI, and SLT.

Participants subsequently attended a face-to-face simulation workshop. During this session, residents practiced each laser procedure using ophthalmic training model eyes (SimulEYE^®^, insEYEt, LLC, Westlake Village, CA, USA) under the direct supervision of consultant ophthalmologists experienced in laser procedures. Dedicated laser machines equipped with external monitors were used to facilitate real-time observation and feedback. Emphasis was placed on correct patient positioning, laser focusing and targeting, appropriate energy titration, and avoidance of common complications.

Confidence was assessed using pre- and post-training questionnaires. Participants rated their confidence in independently performing each procedure using a 10-point Likert scale (1 = not confident at all; 10 = very confident). Confidence scores were recorded separately for YAG capsulotomy, PI, and SLT. Descriptive statistics were used to summarize the data. Pre- and post-training scores were compared using paired Student’s t-tests to assess statistical significance. A p-value < 0.05 was considered statistically significant. Analyses were conducted using GraphPad Prism (GraphPad Software, San Diego, California, USA).

## Results

Forty residents participated in this simulation event, of whom 39 completed both surveys. Thirty-three residents were in their first year of training, with the remainder being in their second year.

**Figure 1:**
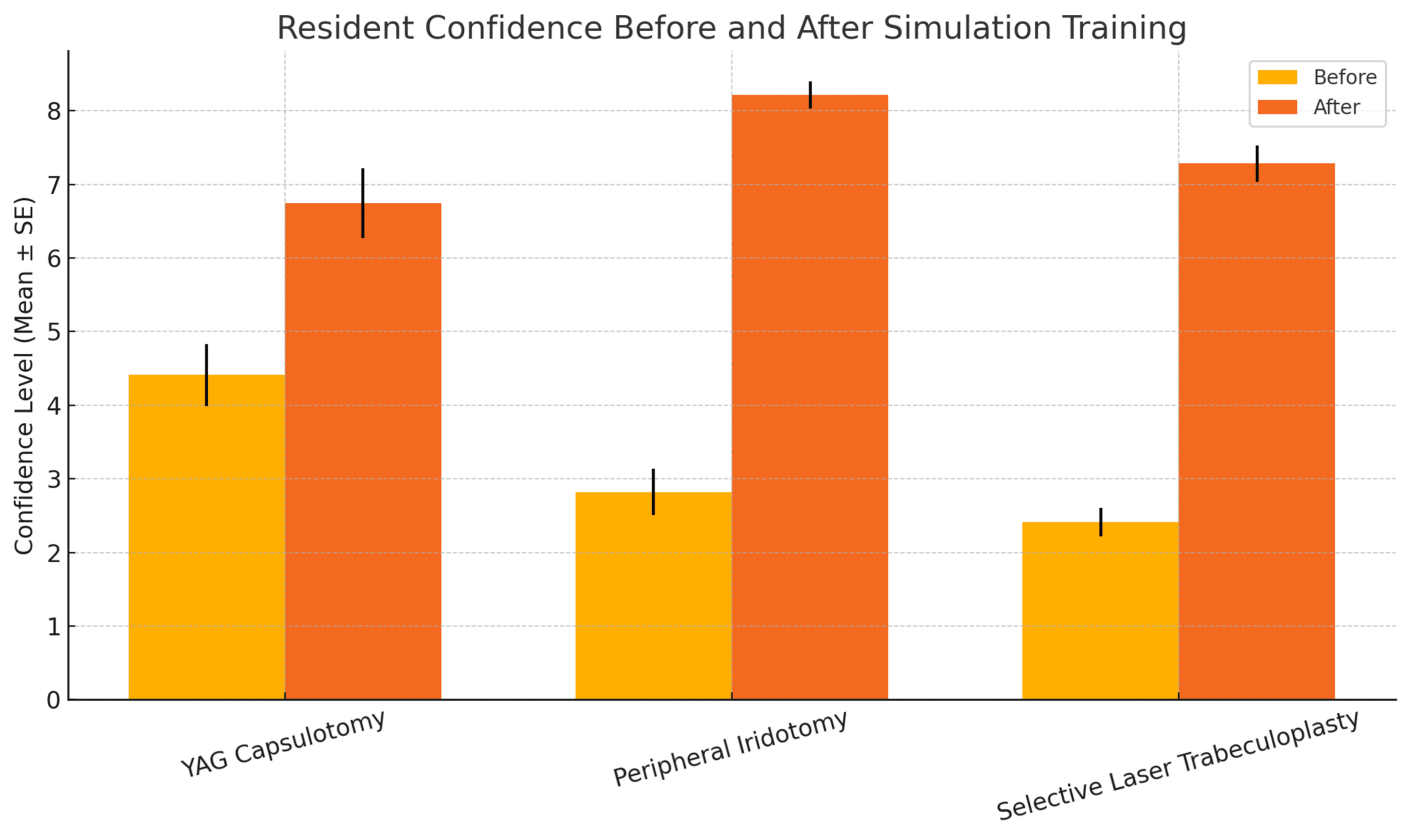
Resident confidence before and after simulation training Mean confidence value (± SE) for each procedure before and after simulation training. YAG, yttrium aluminum garnet

Mean confidence scores increased significantly following the simulation-based training program for all three laser procedures (Figure 1, Table 1). For YAG capsulotomy, mean confidence increased from 4.41 ± 0.42 before training to 6.74 ± 0.48 after training (p < 0.0001, paired Student’s t-test). Confidence in performing PI increased from 2.82 ± 0.32 to 8.21 ± 0.18 (p < 0.0001, paired Student’s t-test). Similarly, confidence in performing SLT increased from 2.41 ± 0.20 to 7.28 ± 0.24 (p < 0.0001, paired Student’s t-test). All residents surveyed expressed a desire for more simulation opportunities to be incorporated into their training.

**Table 1:** Comparison of resident confidence scores before and after simulation training Mean confidence value ± SE for each procedure before and after simulation training. Paired Student’s t-tests were used for comparisons. p < 0.05 was considered statistically significant. PI, peripheral iridotomy; SLT, selective laser trabeculoplasty; YAG, yttrium aluminum garnet

Procedure	Mean ± SE (before)	Mean ± SE (after)	p-Value
YAG capsulotomy	4.41 ± 0.42	6.74 ± 0.48	<0.0001
PI	2.82 ± 0.32	8.21 ± 0.18	<0.0001
SLT	2.41 ± 0.20	7.28 ± 0.24	<0.0001

## Discussion

This study demonstrates that a structured blended learning program, consisting of e-learning modules, instructional videos, and hands-on practice, significantly improves confidence in performing YAG capsulotomy, PI, and SLT among early-stage ophthalmologists. These findings are consistent with prior work, highlighting simulation’s educational value [8]. Montrisuksirikun et al. reported that cataract simulation on model eyes improved both confidence and clinical outcomes, including reduced posterior capsule rupture rates [9]. Similar benefits have been shown for strabismus surgery and emergency procedures such as canthotomy and cantholysis [10,11].

The evaluation of simulation-based training is often guided by established frameworks, with the Kirkpatrick model being one of the most widely applied. This model encompasses four domains: reaction, learning, behavioral change, and real-world outcomes [12]. Our results align with the early stages of the Kirkpatrick model of training evaluation, particularly in the domains of learner reaction and knowledge acquisition [12]. Residents reported high levels of satisfaction with the training and unanimously expressed a desire for additional simulation opportunities. This positive reception highlights both the acceptability and practical feasibility of incorporating blended simulation into ophthalmology training.

While increased confidence is encouraging, further research is necessary to determine whether it translates into meaningful behavioral change and improved patient outcomes, a key indicator in the higher levels of the Kirkpatrick evaluation model. Although this study did not assess clinical performance directly, prior evidence from cataract and emergency procedure simulation has shown reductions in complication rates and improvements in technical skill [9,11]. If similar benefits can be demonstrated for laser procedures, structured simulation could contribute significantly to patient safety. Longitudinal studies will be essential to evaluate these outcomes.

Strengths

This program combined mandatory e-learning, structured assessments, and supervised simulation, ensuring residents consolidated their theoretical understanding before practicing procedural skills. During the structured simulation session, model eyes were used to assess key performance metrics, including laser accuracy, energy use, and safety parameters, while instructors provided real-time feedback. These objective measures reinforced safe clinical behaviors and supported skill development.

Limitations

We primarily assessed self-reported confidence, which may not correlate with real-world competence, performance, or patient outcomes. This limitation has also been noted in a recent systematic review assessing various ophthalmic simulation models [13]. In addition, confidence was assessed immediately following training, making it difficult to evaluate long-term retention, as both technical skills and perceived competence are susceptible to decay over time without continued practice or reinforcement [14]. Resource requirements, including single-use model eyes, laser access, and faculty time, may restrict scalability, particularly in low-resource settings. Innovative, lower-cost alternatives, such as reusable training models, may enhance accessibility. For example, Kong et al. created a low-fidelity, reusable training model to teach lateral canthotomies [15].

## Conclusions

A structured simulation-based training program significantly improved the confidence of ophthalmology residents in performing YAG capsulotomy, PI, and SLT. Residents were given the opportunity to practice in a safe and structured environment with live feedback and guidance. Future studies should assess long-term retention and determine whether increased confidence translates into measurable improvements in patient outcomes.
